# Clinical association analysis of ependymomas and pilocytic astrocytomas reveals elevated FGFR3 and FGFR1 expression in aggressive ependymomas

**DOI:** 10.1186/s12885-017-3274-9

**Published:** 2017-05-03

**Authors:** Birgitta Lehtinen, Annina Raita, Juha Kesseli, Matti Annala, Kristiina Nordfors, Olli Yli-Harja, Wei Zhang, Tapio Visakorpi, Matti Nykter, Hannu Haapasalo, Kirsi J. Granberg

**Affiliations:** 1 BioMediTech Institute and Faculty of Medicine and Life Sciences, Biokatu 8, 33520 Tampere, Finland; 2 Fimlab Laboratories Ltd., Tampere University Hospital, Biokatu 4, 33520 Tampere, Finland; 3 Department of Pathology, University of Tampere, 33014 Tampere, Finland; 4 Department of Pediatrics, Tampere University Hospital; Tampere Center for Child Health Research, University of Tampere, 33014 Tampere, Finland; 5 Department of Signal Processing, Tampere University of Technology, Korkeakoulunkatu 10, 33720 Tampere, Finland; 6 Department of Cancer Biology, Comprehensive Cancer Center of Wake Forest Baptist Medical Center, 1 Medical Center Blvd, Winston-Salem, NC 27157 USA; 7 Science Center, Tampere University Hospital, Biokatu 6, 33520 Tampere, Finland

**Keywords:** Tissue microarray, Deep-sequencing, FGFR inhibition, Immunohistochemistry staining

## Abstract

**Background:**

Fibroblast growth factor receptors (FGFRs) are well-known proto-oncogenes in several human malignancies and are currently therapeutically targeted in clinical trials. Among glioma subtypes, activating FGFR1 alterations have been observed in a subpopulation of pilocytic astrocytomas while FGFR3 fusions occur in IDH wild-type diffuse gliomas, resulting in high FGFR3 protein expression. The purpose of this study was to associate FGFR1 and FGFR3 protein levels with clinical features and genetic alterations in ependymoma and pilocytic astrocytoma.

**Methods:**

FGFR1 and FGFR3 expression levels were detected in ependymoma and pilocytic astrocytoma tissues using immunohistochemistry. Selected cases were further analyzed using targeted sequencing.

**Results:**

Expression of both FGFR1 and FGFR3 varied within all tumor types. In ependymomas, increased FGFR3 or FGFR1 expression was associated with high tumor grade, cerebral location, young patient age, and poor prognosis. Moderate-to-strong expression of FGFR1 and/or FGFR3 was observed in 76% of cerebral ependymomas. Cases with moderate-to-strong expression of both proteins had poor clinical prognosis. In pilocytic astrocytomas, moderate-to-strong FGFR3 expression was detected predominantly in non-pediatric patients. Targeted sequencing of 12 tumors found no protein-altering mutations or fusions in FGFR1 or FGFR3.

**Conclusions:**

Elevated FGFR3 and FGFR1 protein expression is common in aggressive ependymomas but likely not driven by genetic alterations. Further studies are warranted to evaluate whether ependymoma patients with high FGFR3 and/or FGFR1 expression could benefit from treatment with FGFR inhibitor based therapeutic approaches currently under evaluation in clinical trials.

**Electronic supplementary material:**

The online version of this article (doi:10.1186/s12885-017-3274-9) contains supplementary material, which is available to authorized users.

## Background

Fibroblast growth factor receptors (FGFRs) are a family of receptor tyrosine kinases that are activated in a variety of cancers and have well-established oncogenic properties [1, 2]. Since the discovery of recurrent FGFR gene fusions in glioblastoma [3, 4], FGFR inhibitor based treatment regimens have been viewed as a promising therapeutic option for brain tumors with FGFR alterations. The mechanisms of FGFR activation in brain tumors vary by tumor type, but include oncogenic FGFR3 and FGFR1 fusions, FGFR1 rearrangements, and FGFR1 mutations [2–8]. Moreover, gene fusions appear to be the sole recurrent oncogenic FGFR3 alteration in brain tumors. Although FGFR3 is commonly fused to a transforming acidic coiled-coil-containing protein 3 (TACC3) gene, other fusion partners exist. For example, recurrent FGFR3–BAIAP2L1 fusions have been detected in bladder cancer [9]. Several FGFR inhibitors are currently under pre-clinical and clinical evaluation, and recent reports have shown good treatment responses in FGFR3 fusion positive cells and tumors [8, 10, 11]. While most of the FGFR inhibitor studies, to date, have been performed in cases involving carcinomas, responses to FGFR inhibitors have also been reported in cases with glioblastoma [8, 12].

Ependymomas and pilocytic astrocytomas are nondiffuse gliomas, in which neoplastic cells do not substantially infiltrate into surrounding normal tissue. They represent different grades, types of growth and clinical courses. Nondiffuse growth pattern facilitates efficient surgical removal of the tumor, which partly explains the better prognosis in these patients relative to those with diffuse gliomas. However, tumor recurs in some of the patients, and overall survival rates are worse with more aggressive ependymomas [13].

Ependymomas are the third most common brain tumor in children, representing 8–10% of pediatric intracranial tumors and approximately 4% of all adult brain tumors [13]. Ependymomas are found in all locations of the central nervous system, and may be intracranial (infratentorial or supratentorial) or spinal. Infratentorial posterior fossa ependymomas can be further subclassified into posterior fossa group A (PFA) and group B (PFB) tumors [14]. Adult ependymomas are typically grade I myxopapillary ependymomas localized in the spinal cord, while pediatric ependymomas are typically intracranial grade II–III tumors [13, 15]. Although ependymomas in young children are typically associated with poor prognosis [15, 16], adult supratentorial ependymomas are also associated with lower survival rates [13].

Apart from copy number alterations [13], significant genetic and epigenetic drivers of ependymoma development have been recently reported. C11orf95–RELA fusions have been observed to occur in two-thirds of pediatric cases of supratentorial ependymomas and are believed to be oncogenic due to increased NF-kB signaling [17]. Furthermore, a subtype of cerebellar ependymomas that is associated with young patient age and poor prognosis is characterized by a CpG island methylator phenotype (CIMP) and Polycomb repressive complex 2 driven trimethylation of H3K27. These tumors are responsive to pharmacological therapies targeting epigenetic regulators [18]. The authors also highlighted the low rate of recurrent mutations and copy number alterations in cerebellar ependymomas. Furthermore, FGFR alterations have not been reported in high-throughput sequencing studies with the exception of FGFR1 missense mutation N544 K [17] localized to the tyrosine kinase domain of FGFR1.

Pilocytic astrocytoma (PA), the most common brain neoplasm in the pediatric population, is classified as WHO grade I [19, 20]. They arise most commonly in the cerebellum, brainstem and the optic nerve. Familial PAs are characterized by inactivation of the neurofibromatosis 1 (*NF1*) tumor suppressor gene, while activating BRAF fusions and mutations are typical for sporadic PAs [19]. BRAF alterations subsequently lead to activation of the MEK-ERK pathway [19], which is also an important downstream signalling pathway for FGFR-induced signaling [19, 21]. Additionally, FGFR1-TACC1 fusion has been reported in a BRAF wild-type pilocytic astrocytoma of the diencephalon and several studies have reported oncogenic structural FGFR1 variants with duplication of the tyrosine kinase domain [6, 7]. Furthermore, approximately 5% of PAs harbor an FGFR1 mutation targeting codons Asn546 or Lys656 in the kinase domain [7]. The Lys656 mutation has been associated with decreased patient survival [22]. Most FGFR1-mutant tumors studied have been extra-cerebellar, located mostly in midline locations, and mutually exclusive with BRAF, NF1, and other recurrent MAPK pathway alterations [7, 22]. Although these studies did not report mutations or structural variants in FGFR3, they emphasized the utility of FGFR1 as a marker for PA subtyping.

In diffuse gliomas, FGFR3 protein level is an informative marker for fusion status [34]. Most tumors in a cohort of 791 cases did not have any detectable FGFR3 protein expression, and all the fusion-positive cases were strongly stained (staining sensitivity 100% and specificity 88% in the targeted sequencing cohort). In non-diffuse gliomas, FGFR1 alterations are commonly present in a subgroup of pilocytic astrocytomas that lack other typical MAPK pathway alterations [6, 7], but FGFR1 and FGFR3 expression levels have not been systematically evaluated. Futhermore, FGFR fusions or increased FGFR protein expression levels have not, to date, been reported to occur in ependymomas. In the present study, we sought to investigate the clinical significance of FGFR3 and FGFR1 expression in two different nondiffuse gliomas: ependymomas and pilocytic astrocytomas. We used immunohistochemistry to detect FGFR1 and FGFR3 protein levels in ependymomas and pilocytic astrocytomas, and evaluated the relationship between protein expression levels, clinical features and selected genetic alterations.

## Methods

### Patient samples

This study was approved by the Ethical Committee of Tampere University Hospital and the National Authority for Medico-legal Affairs in Finland. The study cohort included 108 ependymal tumors from 88 patients, 97 pilocytic astrocytomas from 97 patients (Table 1).

**Table 1:** Patient demographics and clinical characteristics within ependymoma and pilocytic astrocytoma tumor patient cohorts

	Ependymomas	Pilocytic astrocytomas
Patients	88	80
Male	48	42
Female	40	38
Age (years)		
Median (Mean ± SD)	37 (35 ± 21)	9 (14 ± 14)
Minimum	1	0
Maximum	73	58
Follow-up for primary tumor patients		
Survivors in the end of the follow-up	60	69
Follow-up time for survivors (m) (median (mean ± SD))	125 (135 ± 82)	70 (111 ± 89)
5-year residive-free survival (%)	71	82
5-year survival (%)	82	93
Tumors	108	80
Primary	74	73
Second	14	5
Third	14	1
Fourth-sixth	6	1
Histological grade		
I	18	80
II	68	0
III	22	0
Topography		
Supratentorial	35	3
Infratentorial	28	69
Spinal	43	2
Cranial nerve	0	6

Patient age and follow-up information were calculated using primary cases. Follow-up times are shown in months (m)

*SD* standard deviation

Ependymoma patients underwent neurosurgical operation with the intention of gross radical tumor resection between 1984 and 2009 at Tampere University Hospital, between 1979 and 1998 at Kuopio University Hospital, and between 1986 and 1999 at Turku University Hospital, Finland. The clinical data detail about radicality of tumor resection is imperfect, but radical resection has always been performed when possible for the patient. Grade I tumors included 17 myxopapillary ependymomas and 1 subependymoma. Grade II tumors included 68 ependymomas, while Grade III tumors included 22 anaplastic ependymomas, as classified according to WHO criteria [23].

Pilocytic astrocytoma patients underwent tumor surgery at the Tampere University Hospital between 1985 and 1999, at the Kuopio University Hospital between 1980 and 1992, at the Turku University Hospital between 1981 and 1992, and at the Helsinki University Hospital between 1986 and 1993.

### Tissue histopathology and microarrays

Tumor samples were fixed in formaldehyde (buffered with 4% phosphate) and embedded in paraffin. The samples were processed into paraffin blocks and sections were stained with hematoxylin and eosin (H&E). Histopathological typing and grading, evaluation, and identification of histologically representative tumor regions on each slide were performed by an experienced neuropathologist. Tissue microarray (TMA) blocks were constructed using representative sample regions and a custom-built instrument (Beecher Instruments, Silver Spring, MD, USA). The diameter of the tissue core on the microarray block was 0.6 or 1 mm, depending on the TMA type. Five-micrometer-thick sections were cut from representative array paraffin blocks.

### Immunohistochemistry

Paraffin was removed with hexane. After rehydration in ethanol, the pre-processing stage was performed using Target Retrieval Solution citrate buffer (Dako). The samples were stained using rabbit monoclonal FGFR1 antibody (#9740, Cell Signaling Technology, 1:100 dilution) and mouse monoclonal FGFR3 antibody (sc-13,121, Santa Cruz Biotechnology, 1:600 dilution). ‘Envision + System-horseradish peroxidase and diaminobenzidine (DAB)’ kit (Dako) was used for FGFR3. The nuclei were stained with hematoxylin. A mouse monoclonal antibody MIB-1 (Ki-67 antigen, dilution 1:40, Immunotech, S.A. Marseille, France) was used to analyze cell proliferation. The tissue sections were counterstained with methyl green. The percentage of tissue MIB-1-positive nuclei was quantitatively evaluated using a computer-assisted image analysis system (CAS-200 TM Software, Becton Dickinson & Co., USA) and ImmunoRatio analysis. Only neoplastic cells were included in the analysis (necrotic and hemorrhagic areas were omitted).

The intensity of FGFR3 and FGFR1 immunopositivity was scored by two observers (HH and KG) on a scale from 0 to 3 as follows: 0 (no staining), 1 (weak immunostaining), 2 (moderate immunostaining), or 3 (strong immunostaining).

### Statistical analysis

All data were analyzed using R packages or IBM SPSS statistics 21.0 software (SPSS Inc., Chicago, IL, USA) for Windows. Tests for pairwise association between discrete variables were performed using Fisher’s exact test for count data. For tables larger than 2 × 2, the *p*-values of Fisher’s exact tests were calculated using Monte Carlo simulation with 2.5*10^7 replicates. *p*-values were not corrected for multiple testing. Log-rank test was used for the analysis of prognostic factors. In cox regression analysis, cox model was built using a stepwise forward likehood-ratio testing.

### Targeted sequencing

All the tissue samples were formalin fixed and paraffin embedded (FFPE). A turXTRAC FFPE DNA kit (Covaris) or AllPrep DNA/RNA Mini Kit (Qiagen) was used for DNA isolation. We used 1 μg of extracted DNA for targeted sequencing using the Sureselect XT Target enrichment system together with custom-designed RNA probes (Additional file 1: Table S1). The sequencing library was prepared according to the kit instructions (200 ng of DNA samples) with a shorter DNA-shearing protocol (220 s) and sequenced with MiSeq (Illumina). Tumors Epe002 and Epe003 were derived from the first and the third tumor surgery (after second recurrence) of one patient. In addition, the tumors Epe004 (1st tumor surgery) and Epe005 (2nd tumor surgery) were derived from a separate ependymoma patient.

The resulting data were aligned against the GRCh37 human reference genome using Bowtie 2.2.4 [24]. Mutations were identified in tumor samples by searching for sites with an alternate allele fraction of at least 10%, and at least 5 reads with the mutation. Additionally, the allele fraction was required to be 20 times higher than the background error rate (i.e., the average allele fraction across control blood samples from healthy patients). Protein-level consequences of variants were predicted using ANNOVAR software tool [25]. Mutations with a known or suspected pathological function were identified manually. To discover chromosomal rearrangements for fusion detection, unaligned reads from each sample were split into two 30 bp anchors (one from both ends) that were aligned to the hg38 genome using Bowtie-1.1.2. Discordant anchor pairs were grouped by position, and groups with 8 or more supporting reads were flagged as rearrangement candidates and manually curated using IGV and BLAT.

Log ratios of amplicon read counts were used for DNA copy number calling. Differences in average coverage between samples were corrected on the basis of control amplicons in chromosomes 5, 8, 11, and 18 (14–21 amplicons per chromosome), positioned in regions with the lowest rate of reported copy number alterations. Blood-derived DNA from healthy individuals was used as a negative control for the copy number analysis.

## Results

**Fig. 1:**
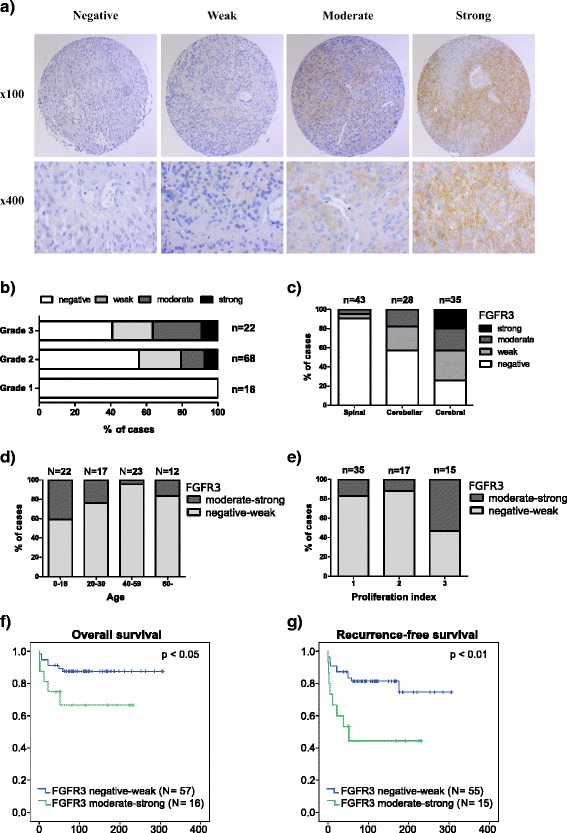
Moderate-to-strong FGFR3 immunostaining was predictive of poor patient survival in ependymomas. **a** Representative staining images. **b** Distribution of FGFR3 immunostaining in grade I–III ependymomas. FGFR3 immunostaining was positively associated with tumor grade (*p* < 0.01, Fisher’s exact test). **c** Moderate-to-strong FGFR3 immunostaining was associated with cerebral tumor location (*p* < 0.0001, Fisher’s exact test). Total number of tumors for each location is marked into the figure. **d** Moderate-to-strong FGFR3 expression was more common in younger patients *(p* < 0.05, Fisher’s exact test). Only newly-diagnosed cases were included in the analysis and these were divided into those with negative-to-weak vs. moderate-to-strong FGFR3 immunostaining. **e** Cases with moderate-to-strong FGFR3 expression tended to have higher proliferation index (*p* = 0.07, Fisher’s exact test). Samples were divided based on FGFR3 staining and proliferation rate (1: low, 2: intermediate, and 3: high proliferation index). **f**-**g** Moderate-to-strong FGFR3 immunostaining was associated with worse g) disease-specific survival (*N* = 73, *p* < 0.05, log-rank test) and g) recurrence-free survival (*N* = 70, *p* < 0.01, log-rank test). Only newly-diagnosed cases were included into the analysis

We used an antibody that targets amino acids 25–124 in the FGFR3 N-terminus to perform immunohistochemical (IHC) staining on 188 cases including ependymomas or pilocytic astrocytomas (Table 1). FGFR3 staining was localized to the cytoplasm and plasma membrane (Fig. 1). Staining was typically heterogeneous in all tumor types studied. Negatively stained blood vessels provided an internal control for antibody specificity. Normal brain tissue was immunonegative, with the exception of the cerebellar and cerebral molecular layers, where weak-to-moderate staining was observed (Additional file 1: Figure S1a).

### In ependymomas, FGFR3 staining is associated with disease aggressiveness

Immunohistochemistry was used to investigate FGFR3 expression levels in 108 ependymal tumor samples applied to TMAs. The TMA cohort (Table 1), representing different grades of ependymomas and disease subtypes, has been partly reported previously [26]. FGFR3 immunoreactivity was detected in 27 (37%) of the cases; 11 (15%) showed weak immunostaining, 11 (15%) showed moderate immunostaining and 5 (7%) were strongly immunopositive. Increased staining was also observed in pseudorosette structures (Additional file 1: Figure S1b). Recurrent tumors showed typically similar staining levels as the primary tumor. With respect to the association analysis (Additional file 1: Figure S2), FGFR3 staining was significantly associated with a higher tumor grade (*p* < 0.01, Fisher’s exact test, Fig. 1b, Table 2). None of the grade I cases showed detectable FGFR3 expression. Moderate-to-strong FGFR3 immunostaining was predominantly detected in cerebral tumors as compared to other locations (*p* < 0.001, Fisher’s exact test, Fig. 1c, Table 2). Elevated FGFR3 immunopositivity in high-grade cerebral tumors suggests that FGFR3 immunostaining may be typical for pediatric ependymomas. Indeed, patients with age < 20 years at tumor onset had a higher frequency of FGFR3 immunopositive staining (*p* < 0.05, Fisher’s exact test, Fig. 1d). Cases with moderate-to-strong FGFR3 immunostaining tend to show a high proliferation rate (Fig. 1e), although this association was not statistically significant (*p* = 0.07, Fisher’s exact test). Importantly, moderate-to-strong FGFR3 immunostaining was significantly associated with shorter overall patient survival (*p* < 0.05, log-rank test, Fig. 1f) and shorter time to tumor recurrence (*p* < 0.01, log-rank test, Fig. 1g). The association with disease-free survival remained significant after adjustment for tumor location, grade, and proliferation (*p* = 0.003, RR = 1.82, 95% CI 1.23–2.68 for FGFR3, other variables not significant in the final equation, *N* = 77, stepwise Cox regression), but only tumor location (*p* = 0.022, RR = 2.47, 95% CI 1.42–5.34, *N* = 77, stepwise Cox regression) was a significant prognostic predictor for disease-specific survival in multifactorial analysis. It is relevant to note the patient numbers (*N* = 77) are rather low for multifactorial analysis using four different variables. Still, the obtained results suggest that FGFR3 immunopositivity is associated with more aggressive ependymomas.

**Table 2:** Samples numbers in FGFR1 low, FGFR1 high, FGFR3 low, and FGFR3 high groups in respect to tumor location, tumor grade and patient age

	FGFR1 low	FGFR1 high	FGFR3 low	FGFR3 high
Tumor location				
Spinal	37	6	41	2
Cerebellar	21	4	23	5
Cerebral	16	20	20	15
*p*-value	0.0001		0.0002	
Tumor grade				
I	16	2	18	0
II	50	15	54	14
III	10	13	14	8
*p*-value	0.002		0.013	
Patient age				
< 16	22	12	23	12
> =16	50	18	61	10
*p*-value	0.15		0.055	

*p*-values have been calculated using Fisher’s exact test. High: Moderate-to-strong immunostaining, Low: Negative-to-low immunostaining

As pediatric and adult ependymomas differ in many respects and the age association might influence the observed associations, we analyzed the pediatric and adult sample cohorts independently. Patients that were at least 16 years old were considered as adults according to general practice in Finnish pediatric clinics. There were 35 pediatric and 73 adult samples in our cohort. Moderate-to-strong FGFR3 staining was slightly more common in pediatric than adult samples (34.3% vs 13.7%, *p* = 0.055, Fisher’s exact test, Table 2). In pediatric patients, moderate FGFR3 immunostaining was observed in cerebellar (31%, *n* = 16) and cerebral (29%, *n* = 14) tumors and strong FGFR3 staining only in cerebral tumors (21%, *n* = 14), whereas all the spinal cases (*n* = 5) were negative for FGFR3 (*p* = 0.065, Fisher’s exact test). FGFR3 staining was not associated with tumor grade or proliferation index in pediatric ependymomas. In adults, FGFR3 associations were largely very similar as in the whole sample cohort: stronger FGFR3 staining was associated with tumor grade (*p* < 0.01, *n* = 73, Fisher’s exact test), tumor location (*p* < 0.001, *n* = 71, Fisher’s exact test) and there was a close-to-significant association with proliferation index (*p* = 0.095, *n* = 66, Fisher’s exact test). Prognostic associations were mostly nonsignificant in separate survival analyses in pediatric (*n* = 14) and adult (*n* = 30) sample cohorts, but this was likely due to low sample count in the analysis, as the trend remain the similar. Of note, when FGFR3 staining was divided into four groups, it was associated with worse disease-specific (*p* < 0.01, log-rank test) and disease-free (*p* < 0.001, log-rank test) survival in pediatric patients.

### FGFR1 staining is associated with higher tumor grade and cerebral location

The interpretation of the FGFR1 immunostaining data was not as straightforward as FGFR3 staining, partly because macrophages, neurons, and necrotic areas showed immunopositive staining. Therefore, FGFR1 immunohistochemical scoring was based on the presence of FGFR1-positive malignant cell clusters or larger tumor areas (i.e. diffuse staining), and scoring of individual cells was omitted in the analysis. Sporadic moderate-to-strong FGFR1 immunopositivity was also detected and characterized by high outlier expression in individual malignant cells. These observations support those from previous reports [27]. FGFR1 staining was detected in the cytoplasm and membrane compartments, while granular staining was also observed in a subpopulation of positively-stained samples. Interestingly, moderate-to-strong FGFR1 immunostaining was also observed in ependymal rosettes (Additional file 1: Figure S3).

**Fig. 2:**
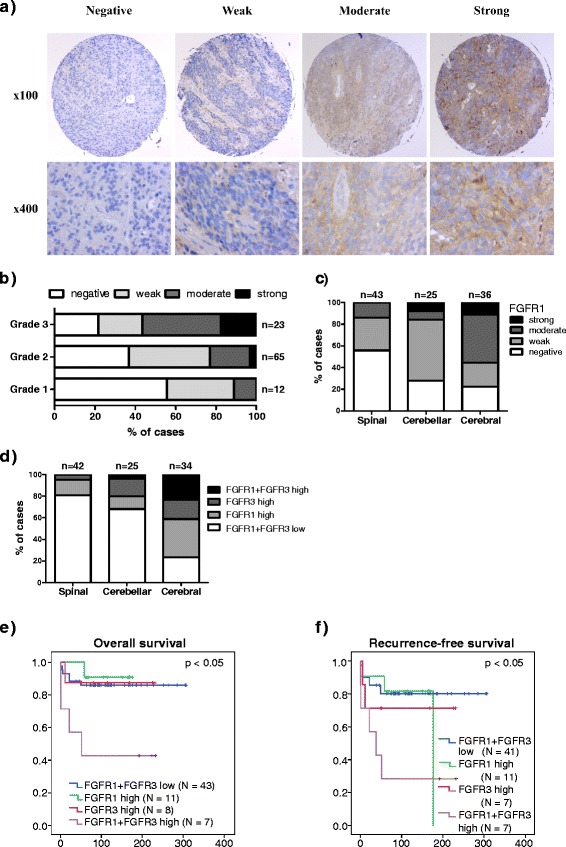
Moderate-to-strong FGFR1 and/or FGFR3 expression is characteristic of aggressive ependymomas. **a** Representative images for FGFR1 staining in ependymomas. **b** The distribution of FGFR1 immunostaining in grade I-III ependymomas. FGFR1 staining was associated with higher tumor grade (*p* < 0.05, Fisher’s exact test). **c** Moderate-to-strong FGFR1 immunostaining was associated with cerebral tumor location (*p* < 0.01, Fisher’s exact test). Total number of tumors for each location is marked into the figure. **d** Moderate-to-strong immunostaining of FGFR1 and/or FGFR3 was detected in a majority of cerebral ependymoma samples (*p* < 0.0001, Fisher’s exact test). **e**-**f**). Moderate-to-strong immunostaining of both FGFR3 and FGFR1 was associated with e) poor disease-specific survival (*N* = 69, *p* < 0.05, log-rank test) and **f** worse recurrence-free survival (*N* = 66, *p* < 0.05, log-rank test). Newly diagnosed cases were divided into four categories based on the expression of both FGFR1 and FGFR3. High: Moderate-to-strong immunostaining, Low: Negative-to-low immunostaining

Diffuse FGFR1 immunoreactivity was detected in 42 (58%) of ependymal tumors. Twenty-four cases (33%) showed weak immunostaining, 15 (21%) cases showed moderate immunoreactivity, and 3 (4%) cases showed strong immunopositivity (Fig. 2a). Consistent with FGFR3 expression, FGFR1 immunostaining was significantly associated with a higher tumor grade (*p* < 0.05, Fisher’s exact test, Fig. 2b, Table 2) and cerebral location (*p* < 0.01, Fisher’s exact test, Fig. 2c, Table 2). Diffuse FGFR1 staining was not significantly associated with overall or recurrence-free survival but cases with high FGFR1 expression had a tendency toward decreased survival rates in this cohort (Additional file 1: Figure S4). When ependymomas were divided into pediatric (*n* = 34) and adult (*n* = 72) patients, no associations were observed for FGFR1 in the pediatric cohort. However, FGFR1 staining was similarly associated with tumor location (*p* < 0.001, *n* = 70, Fisher’s exact test) and higher tumor grade (*p* < 0.01, *n* = 72, Fisher’s exact test) in the adult cohort as in the whole sample cohort. Furthermore, a weak association was observed between stronger FGFR1 staining and higher tumor proliferation index (*p* = 0.061, *n* = 68, Fisher’s exact test) among adult patients.

### FGFR1 and/or FGFR3 levels are elevated in majority of the cerebral ependymomas

Among ependymomas, marked (moderate-to-strong) immunostaining for FGFR1, FGFR3, or both proteins occurred more frequently in cerebral than in non-cerebral tumors (76, 32, and 19% in cerebral, cerebellar, and spinal tumors, respectively, *p* < 0.001, Fisher’s exact test, Fig. 2d). Increased FGFR1 and/or FGFR3 expression was therefore a common characteristic of cerebral tumors. Strikingly, tumor tissues expressing marked (moderate-to-strong) levels of both FGFR1 and FGFR3 were associated with significantly worse patient survival than tissues obtained from other cases, in terms of both overall mortality (*p* < 0.05, log-rank test, Fig. 2e) and recurrence-free survival (*p* < 0.05, log-rank test, Fig. 2f). Furthermore, the combined variable for FGFR1 and FGFR3 (both are negative-to weak, either staining is moderate-to-strong or both are moderate-to-strong) was the only significant predictor for the disease-specific survival (*p* = 0.014, RR = 1.91, 95% CI 1.14–3.20, *N* = 77, stepwise Cox regression) and disease-free survival (*p* = 0.007, RR = 1.75, 95% CI 1.17–2.62, *N* = 77, stepwise Cox regression), when it was combined together with tumor location, grade, and proliferation index as explanatory factors in the multifactorial analysis. It is good to remember that the patient numbers (*N* = 77) are rather low for multifactorial analysis using four different variables when interpreting these results. Still, the obtained results support the aggressive nature of tumors with moderate-to-strong staining of both FGFR1 and FGFR3. Our results are also concordant with previous notions (e.g. [28]) that supratentorial and infratentorial ependymomas are largely different and appear to represent distinct tumor entities.

### FGFR3 staining is associated with increased patient age in pilocytic astrocytoma

**Fig. 3:**
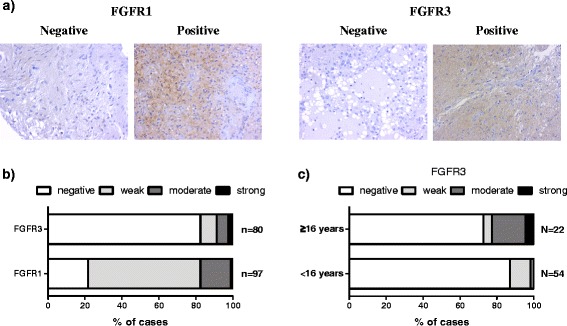
FGFR3 and FGFR1 staining in pilocytic astrocytoma. **a** Representative immunohistochemical images in pilocytic astrocytoma. **b** Distribution of immunohistochemistry scores. The majority of samples were negative for FGFR3. **c** Nearly all of the pilocytic astrocytoma samples showing moderate-to-strong FGFR3 immunostaining were obtained from non-pediatric patients (*p* < 0.01, Fisher’s exact test). Only newly-diagnosed tumors were included into this analysis

In the pilocytic astrocytoma cohort, 60 (82%) samples were negative for FGFR3 expression, while only 21 cases (22%) failed to show any FGFR1 expression (Fig. 3c-d). Among samples with FGFR3 immunoreactivity, 7 samples (9%) showed weak immunostaining, 5 samples (6%) showed moderate immunostaining, and 2 samples (3%) were strongly immunopositive. Immunopositive FGFR3 staining was detected in both microcystic and pilocytic areas. Among samples with positive FGFR1 staining, 59 samples (61%) showed weak immunopositivity, 16 samples (16%) samples showed moderate immunopositivity, and 1 sample (1%) was strongly immunopositive. Moderate-to-strong FGFR1 immunostaining was detected predominantly in microcystic areas. Clinical association analysis (Additional file 1: Figure S5) did not reveal any significant associations between FGFR1 staining and other clinical factors. Interestingly, moderate-to-strong FGFR3 protein levels were associated with increased patient age (≥16 years, *p* < 0.01, Fisher’s exact test, Fig. 3e). All but one of the six primary cases showing moderate-to-strong FGFR3 immunostaining were from patients who were at least 15 years old. FGFR3 immunostaining was not associated with tumor location or aneuploidy.

### Absence of FGFR1 or FGFR3 fusions in targeted sequencing cohort

**Fig. 4:**
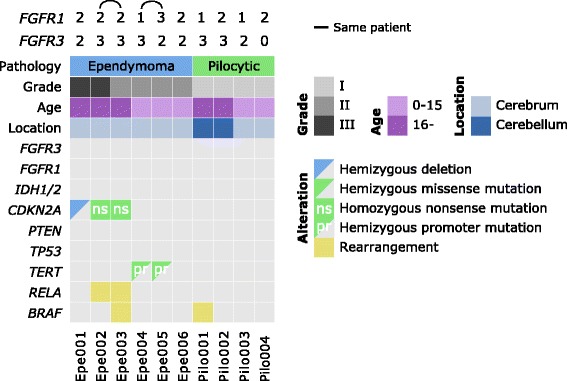
Summary of genetic alterations in the cases that were analyzed using targeted sequencing. No coding mutations or gene fusions were detected in FGFR3 or FGFR1. FGFR1 and FGFR3 immunohistochemical staining scores are shown above the figure. If stained whole-mount tissue slides were available, they were used for scoring. Pilocytic: pilocytic astrocytoma

Ten tumors showing moderate-to-strong FGFR1 or FGFR3 immunostaining were selected for targeted sequencing analysis. All analyzed ependymomas were supratentorial. In addition to FGFR3 and FGFR1, the sequencing panel incorporated genes with reported alterations in gliomas, including *IDH1, IDH2*, *TP53*, *ATRX*, *CIC*, *CDKN2A, RB1, RELA,* and *BRAF* (Additional file 1: Table S1). We did not detect FGFR coding mutations or fusions in any of the samples (Fig. 4, Additional file 2: Table S2, Additional file 1: Figure S6). FGFR3 fusions were detected with high sensitivity from large diffuse glioma cohort using the same sequencing panel and methodology [34], suggesting that the lack of detectable FGFR fusions was not due to methodological limitations. The tumors selected for analysis contained many known alterations, including a *C11orf95-RELA* fusion and *CDKN2A* alterations in ependymoma tumors (Epe001, Epe002 and Epe003). RELA fusions and loss of *CDKN2A* have been routinely observed in aggressive ependymomas [17, 29, 30]. A *TERT* promoter mutation was observed in tumors Epe004 and Epe005 obtained from the same ependymoma patient. In addition, one pilocytic astrocytoma tumor harbored the KIAA1549-BRAF fusion, which is the most frequent MAPK pathway alteration in this tumor type [7]. It is interesting that majority of sequenced PA samples did not carry any BRAF or FGFR1 alterations, but limited sample size does not allow full generalization of this result. A total of 4 cases in our cohort did not carry any alterations in targeted genes. This may be due, in part, to the fact that all genomic regions were not covered during targeted sequencing. In addition, pilocytic astrocytomas are known to harbor very few alterations [7].

## Discussion

Our results demonstrate that moderate-to-strong FGFR3 and/or FGFR1 immunostaining was detectable in most of the supratentorial ependymomas. In ependymoma, moderate-to-strong FGFR3 staining was associated with tumor location, higher proliferation index, and higher grade. Similar associations were obtained when only adult patients were included into the analysis. Moderate-to-strong FGFR3 staining was more frequently observed among pediatric patients than among adults, but only the association between FGFR3 and tumor location remained significant in the pediatric cohort. This might be partly due to a small number of pediatric cases (*n* = 35) and shortage of grade I tumors (*n* = 1) among children. In any case, the data suggest that clinical associations for FGFR3 were not solely due to age-related differences. The situation was similar for FGFR1: moderate-to-strong staining was associated with tumor location and higher grade in both the whole and the adult cohort, despite the lack of clinical associations in the pediatric cohort.

Tumors with high expression of both FGFR3 and FGFR1 were associated with poor clinical prognosis in ependymoma, suggesting that aggressive supratentorial ependymomas may benefit from treatment regimens based on FGFR inhibition. Additional work is required to elucidate the significance of high FGFR1 and/or FGFR3 expression as independent prognostic factors for treatment response. The absence of FGFR alterations in these tumors does not rule out the possibility of treatment response. In head and neck squamous cell cancers and various lung cancers, FGFR1 expression has, in fact, been shown to predict treatment responses better than genomic alterations in FGFR1 [31, 32]. The location of ependymal tumors may also permit drug delivery directly via the cerebrospinal fluid, which would make the treatment less systemic. Many traditional FGFR inhibitors target also other growth factor receptors, such as VEGFR and PDGFR [10], which might also be beneficial. For example, Sie et al. [33] have shown that low grade astrocytoma and ependymoma cell viability decreased upon the single use of one inhibitor on VEGF, EGF, HGF, FGF and PDGF in vitro. On the other hand, the recently developed FGFR-specific inhibitors have generated responses in patients carrying FGFR alterations and are typically associated with less toxic side effects [12], which makes them a favorable treatment option for these patients.

In pilocytic astrocytoma, moderate-to-strong FGFR3 staining was mostly observed in adult patients, which is opposite to the trend in ependymoma, where moderate-to-strong FGFR3 staining was more frequent in pediatric cases. This further suggests that higher FGFR3 expression is not directly linked to young patient age or pediatric tumor type.

In the present study, we did not detect any FGFR fusions or coding mutations in the targeted sequencing cohort. An FGFR1 Lys656 mutation has been reported to occur in the absence of detectable FGFR1 expression in PA [22], suggesting that immunohistochemical data may serve as a valuable prognostic marker when FGFR inhibition is considered as a therapeutic option. FGFR1 is recurrently altered in PA but only in a minority of cases, and, to date, the presence of FGFR1 Lys656 mutation has not been shown to correlate with FGFR1 staining intensity [22]. Intracranial FGFR3 gene fusions have been only detected in IDH wild-type diffuse gliomas [4, 6, 8, 34], suggesting that FGFR3 fusions may contribute to the characteristics of this highly aggressive and invasive type of glioma. We have previously reported that FGFR3 fusion-positive cells were highly invasive and predictive of poor prognosis in a xenograft model [3]. Although FGFR1 fusions are rare in glioma, one fusion-positive pediatric pilocytic/pilomyxoid astrocytoma case has been previously reported [6], suggesting that FGFR1-fusions are not restricted to diffuse gliomas. Moreover, various FGFR1 alterations have been observed in pilocytic astrocytomas [6, 7], suggesting that genetic FGFR1 alterations do not necessarily drive the development or progression of highly malignant tumors.

Despite the high structural similarity between endogenous FGFR1 and FGFR3, these results indicate that functional differences may exist between the altered proteins. Although the clinical associations of FGFR1 and FGFR3 immunostaining showed striking similarities, associations between protein expression and patient survival were significant only for FGFR3. These observations may be related to the relatively small cohort size (approximately 70 primary cases) involved in the present study. The difficulty in interpreting FGFR1 immunostaining, combined with the lack of a significant survival association in our cohort, suggests that FGFR1 staining may not be as useful for patient stratification as FGFR3.

Majority of cases did not show any detectable FGFR3 in both tumor types, which is consistent with our previous results [34]. However, the proportion of patients with moderate-to-strong FGFR3 immunostaining was higher in ependymoma when compared to the diffuse astrocytoma patient cohort (5%, [34] or pilocytic astrocytoma (9%). Since FGFR3 fusions were not detected in any tumors in this study, increased FGFR3 levels are likely to be caused by differences in the *trans*-acting regulation of protein expression.

## Conclusions

Fibroblast growth factors are well-known oncogenes, which have also been targeted in clinical trials. This study reports variable FGFR1 and FGFR3 protein levels in ependymoma and pilocytic astrocytoma. In ependymoma, moderate-to-strong expression of FGFR3 was associated with cerebral location, young patient age and poor prognosis. Ependymoma cases that co-expressed moderate-to-strong levels of both FGFR3 and FGFR1 had significantly lower survival rates. In pilocytic astrocytoma, moderate-to-strong FGFR3 staining was observed predominantly in non-pediatric patients. Targeted sequencing analysis did not detect any coding alterations in *FGFR1* or *FGFR3* genes in staining-positive cases. This is different in diffuse gliomas, were strong FGFR3 staining can be used to indicate the presence of FGFR3 fusion. However, FGFR inhibition might be a suitable treatment option for ependymomas with moderate-to-strong FGFR3 or FGFR3 + FGFR1 expression, as these patients had poor prognosis and we are currently lacking efficient regimens for their treatment.

## Additional files

Additional file 1: Supplementary Material. Description of data: **Table S1.** Target regions for probe design for targeted sequencing. Coordinates were extracted using genome assembly GRCh37/hg19. **Figure S1.** Representative staining images. a) Weak-to-moderate FGFR3 staining was observed in cerebellar molecular layer (100× magnification). b) FGFR3 staining in pseudorosette structures in ependymoma (200× magnification). **Figure S2.** Association analyses in the ependymoma cohort including all the cases. *p*-values were calculated using Fisher’s exact test. **Figure S3.** Moderate-to-strong FGFR1 staining in ependymal rosettes. **Figure S4.** Survival association analysis for FGFR1 staining in the ependymoma cohort was not statistically significant. a) Overall survival, b) Recurrence-free survival. Newly diagnosed cases were divided into two categories: low (negative-to-weak) or high (moderate-to-strong) FGFR1 staining. **Figure S5.** Association analyses in the pilocytic astrocytoma cohort including all the cases. *p*-values were calculated using Fisher’s exact test. **Figure S6.** Alignment and coverage statistics of the targeted sequencing cohort. (a) Total reads, grouped by alignment result. (b) Number of duplicate reads among all aligned reads. (c) Violin plot showing coverage distribution across all bases in target regions. (PDF 6130 kb)

Additional file 2: Table S2. Description of data: Overview of targeted sequencing cohort and the obtained results. (XLSX 101 kb)

## Abbreviations

CIMP: CpG island methylator phenotype
FFPE: Formalin fixed and paraffin embedded
FGFR: Fibroblast growth factor receptor
H&E: Hematoxylin and eosin
IHC: Immunohistochemical
NF1: Neurofibromatosis 1
PA: Pilocytic astrocytoma
TACC3: Transforming acidic coiled-coil-containing protein 3
TMA: Tissue microarray
