# Supplementary material for: Clinical association analysis of ependymomas and pilocytic astrocytomas reveals elevated FGFR3 and FGFR1 expression in aggressive ependymomas
Source: BMC Cancer. 2017 May 3;17:310. doi: 10.1186/s12885-017-3274-9 (PMC5415775; doi:10.1186/s12885-017-3274-9)
Supplement: Supplementary file 1 — Supplementary Material. Description of data: Table S1. Target regions for probe design for targeted sequencing. Coordinates were extracted using genome assembly GRCh37/hg19. Figure S1. Representative staining images. a) Weak-to-moderate FGFR3 staining was observed in cerebellar molecular layer (100× magnification). b) FGFR3 staining in pseudorosette structures in ependymoma (200× magnification). Figure S2. Association analyses in the ependymoma cohort including all the cases. p-values were calculated using Fisher’s exact test. Figure S3. Moderate-to-strong FGFR1 staining in ependymal rosettes. Figure S4. Survival association analysis for FGFR1 staining in the ependymoma cohort was not statistically significant. a) Overall survival, b) Recurrence-free survival. Newly diagnosed cases were divided into two categories: low (negative-to-weak) or high (moderate-to-strong) FGFR1 staining. Figure S5. Association analyses in the pilocytic astrocytoma cohort including all the cases. p-values were calculated using Fisher’s exact test. Figure S6. Alignment and coverage statistics of the targeted sequencing cohort. (a) Total reads, grouped by alignment result. (b) Number of duplicate reads among all aligned reads. (c) Violin plot showing coverage distribution across all bases in target regions. (PDF 6130 kb) [file 12885_2017_3274_MOESM1_ESM.pdf]

[illegible]

Supplementary Table S1. Target regions for probe design for targeted sequencing. Coordinates were extracted using genome assembly GRCh37/hg19.

**Genomic region**	**Target**
chr4:1,790,039-1,820,600	FGFR3 locus
chr8:38,258,656-38,331,352	FGFR1 locus
chr5:1,295,162-1,295,662	TERT promoter (500 bp)
chr13:48,877,883-49,056,026	RB1 exons
chr21:17,911,328-17,911,570	miR-99a locus
chr9:8,314,246-10,612,723	PTPRD exons
chrX:76,937,000-76,940,142	ATRX # exon 8
chrX: 76,763,788-76,891,418	ATRX # exons 16-end
chr8:38,585,704-38,710,546	TACC1 exons
chr4:1,723,217-1,746,905	TACC3 exons
chr9:21,967,751-21,994,490	CDKN2A exons
chr10:89,623,195-89,728,532	PTEN exons
chr3:178,916,614-178,952,152	PIK3CA exons in coding region
chr12:112,856,536-112,947,717	PTPN11 exons
chr4:55,095,264-55,146,925	PDGFRA exons
chr4:55,095,264-55,146,925	MET exons
chr17:7579650-7579750	P53 # exon 3
chr17:7579300-7579600	P53 # exon 4
chr17:7578350-7578570	P53 # exon 5
chr17:7578150-7578300	P53 # exon 6
chr17:7577480-7577630	P53 # exon 7
chr17:7577000-7577180	P53 # exon 8
chr17:7576840-7576940	P53 # exon 9
chr17:7576520-7576680	P53 # exon 10
chr17:7573910-7574050	P53 # exon 11
chr2:209113040-209113190	IDH1 # exon 4
chr15:90631790-90631940	IDH2 # exon 4
chr11:65429530-65431330	RELA # exons 1-3
chr7:140453090-140453190	BRAF # exon 15
chr7:140481400-140494300	BRAF # exons 8-11
chr7:55084974-55275667	EGFR gene
chr5: 60003061 -80002241	CNA negative control region 1 (21 probes)
chr8: 60001061 -80002241	CNA negative control region 2 (21 probes)
chr18: 30000975 -50001334	CNA negative control region 3 (14 probes)
chr11: 70001061-90005243	CNA negative control region 4 (19 probes)

Supplementary Table 2. Overview of targeted sequencing cohort and the obtained results (separate file)

a)

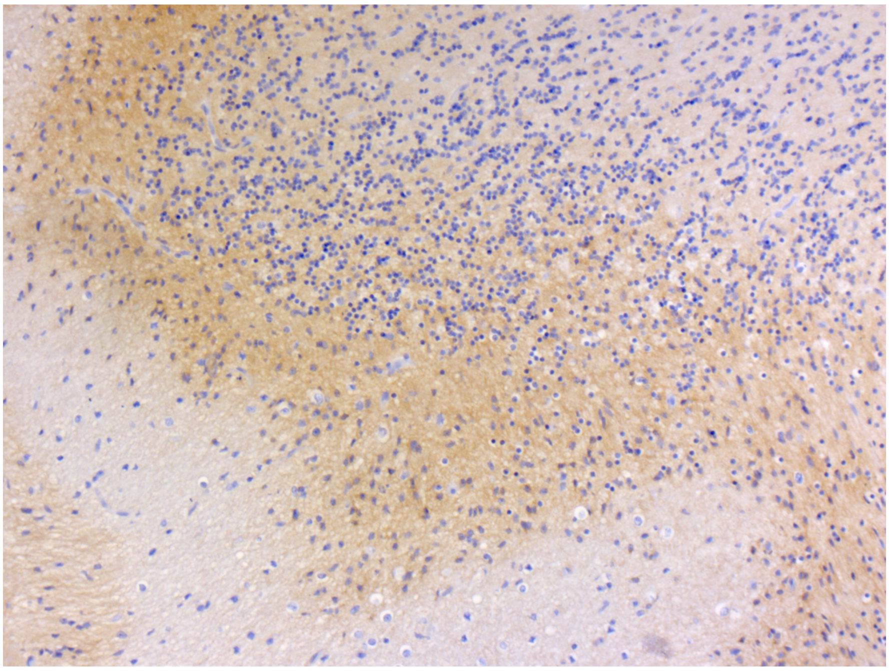

b)

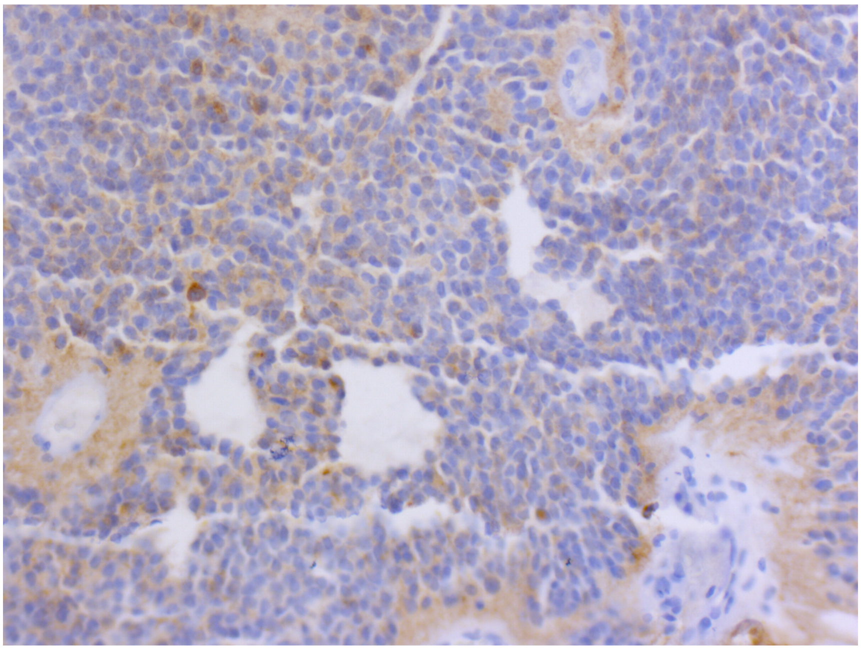

Supplementary Figure S1. a) Weak-to-moderate FGFR3 staining was observed in cerebellar molecular layer (100x magnification). b) FGFR3 staining in pseudorosette structures in ependymoma (200x magnification).

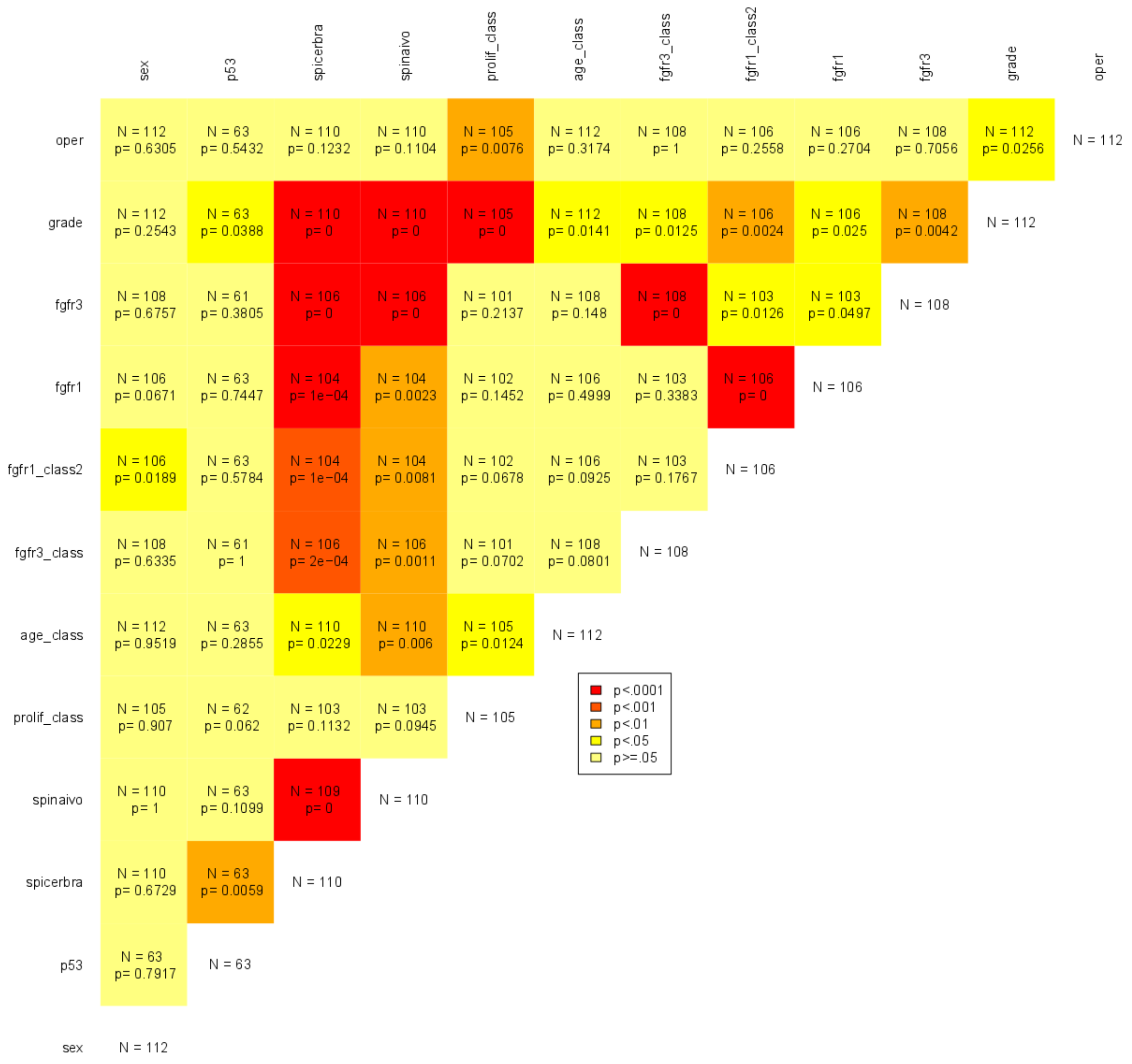

Supplementary Figure {2. Association analyses in the ependymoma cohort including all the cases. p-values were calculated using Fisher's exact test. Cut-off points for patient age classes were 20, 40, 60, and 80 years. In FGFR1 and FGFR3 class, cases were separated into high (moderate or strong staining) and low (negative or weak staining) classes. Oper: primary or recurrent tumor, p53: p53 IHC staining, spicerbra: tumor location (spinal, cerebellum, cerebrum), spinaivo: tumor location (spinal, brain), prolif_class: proliferation index.

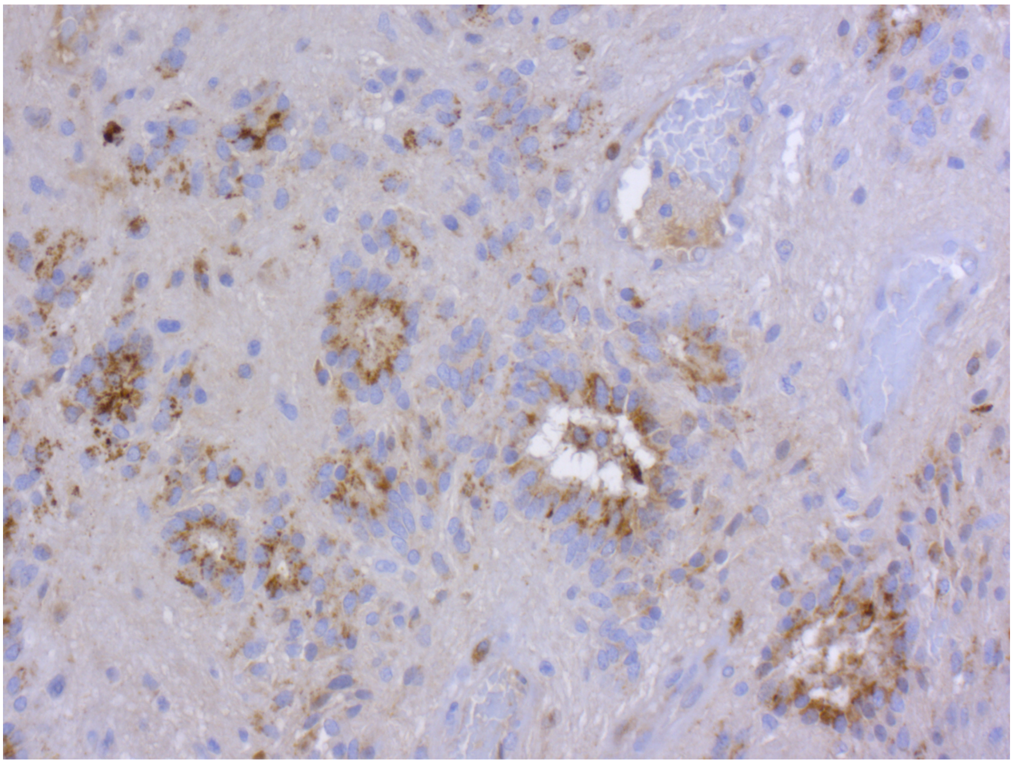

Supplementary Figure S3. Moderate-to-strong FGFR1 staining in ependymal rosettes.

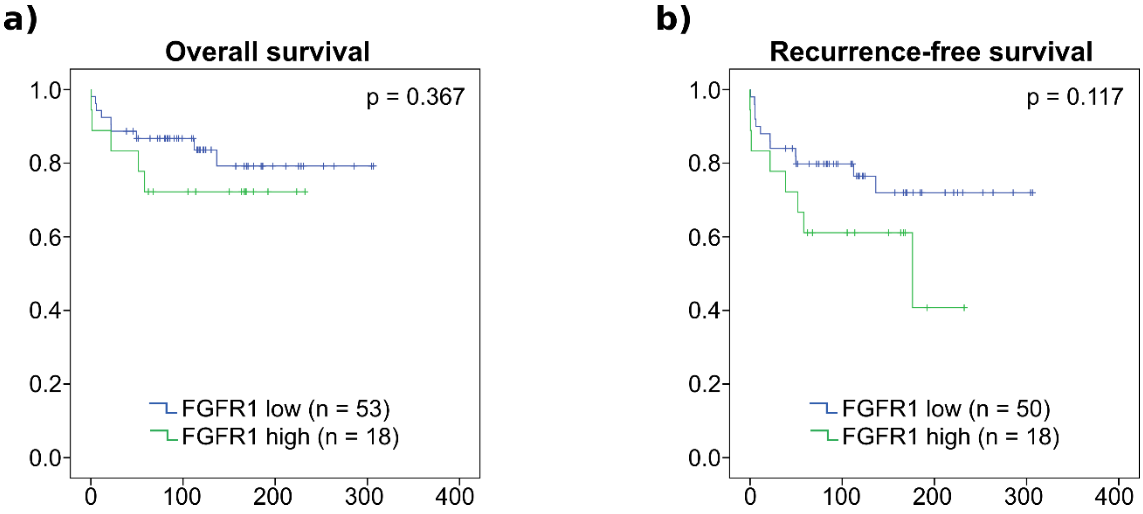

Supplementary Figure S4. Survival association analysis for FGFR1 staining in the ependymoma cohort was not statistically significant. a) Overall survival, b) Recurrence-free survival. Newly diagnosed cases were divided into two categories: low (negative-to-weak) or high (moderate-to-strong) FGFR1 staining.

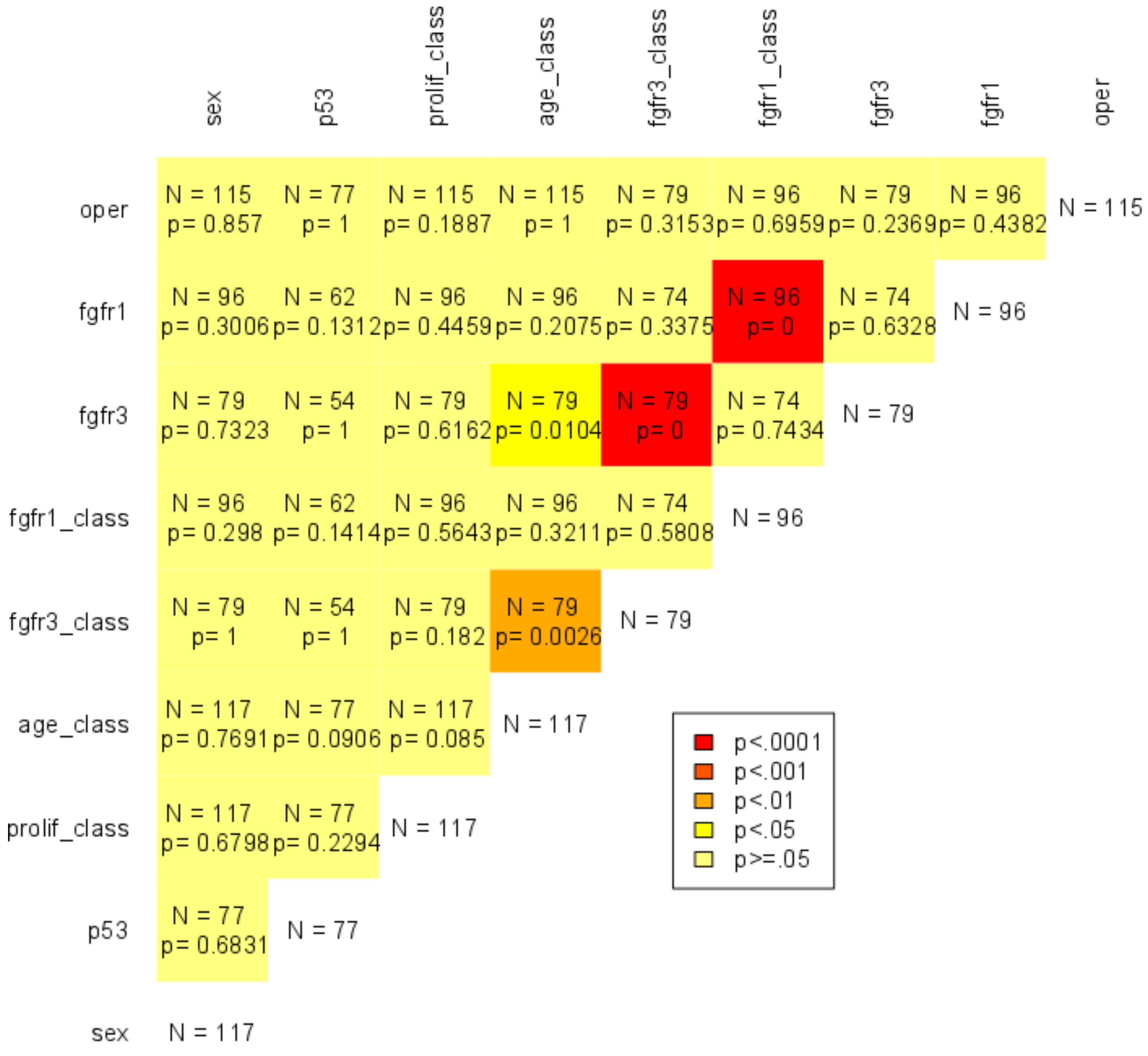

Supplementary Figure S5. Association analyses in the pilocytic astrocytoma cohort including all the cases. p-values were calculated using Fisher's exact test. Cut-off points for patient age classes were 20, 40, 60, and 80 years. In FGFR1 and FGFR3 class, cases were separated into high (moderate or strong staining) and low (negative or weak staining) classes. Oper: primary or recurrent tumor, p53: p53 IHC staining, prolif_class: proliferation index.

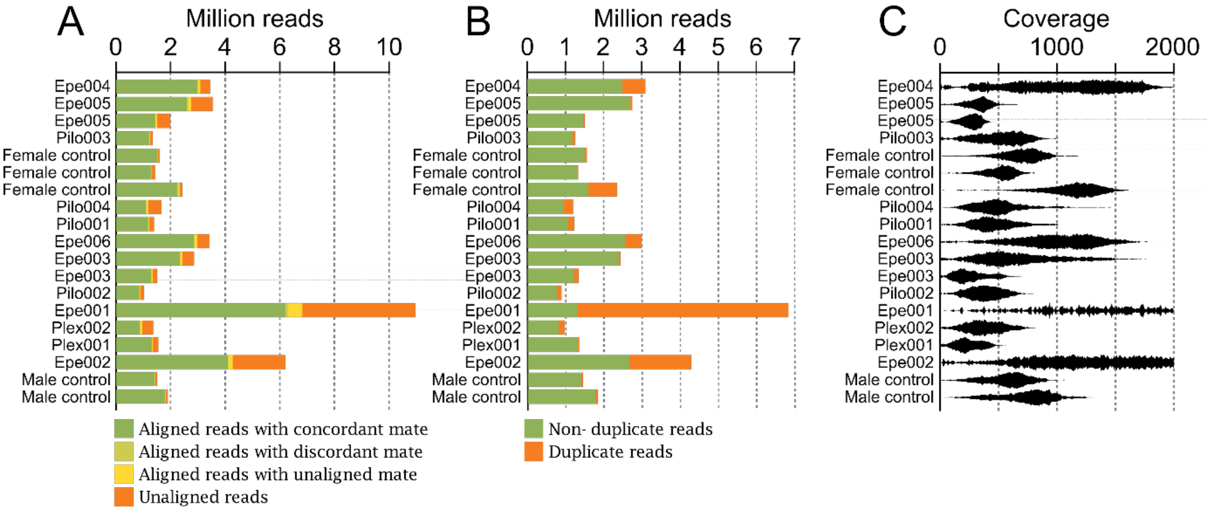

Supplementary Figure S6. Alignment and coverage statistics of the targeted sequencing cohort. (a) Total reads, grouped by alignment result. (b) Number of duplicate reads among all aligned reads. (c) Violin plot showing coverage distribution across all bases in target regions.
